# Evolution and expression of BMP genes in flies

**DOI:** 10.1007/s00427-013-0445-9

**Published:** 2013-04-18

**Authors:** Karl R. Wotton, Anna Alcaine Colet, Johannes Jaeger, Eva Jimenez-Guri

**Affiliations:** 1 EMBL/CRG Systems Biology Research Unit, Centre for Genomic Regulation (CRG), Dr. Aiguader 88, 08003 Barcelona, Spain; 2 Universitat Pompeu Fabra (UPF), Dr. Aiguader 88, 08003 Barcelona, Spain

**Keywords:** Bone morphogenetic proteins (BMPs), Phylogenetic analysis, Gene duplication, Diptera, *Clogmia albipunctata*

## Abstract

Bone morphogenetic proteins (BMPs) play key roles in development. In *Drosophila melanogaster*, there are three BMP-encoding genes: *decapentaplegic* (*dpp*), *glass bottom boat* (*gbb*) and *screw* (*scw*). *dpp* and *gbb* are found in all groups of insects. In contrast, the origin of *scw* via duplication of an ancestral *gbb* homologue is more recent, with new evidence placing it within the Diptera. Recent studies show that *scw* appeared basal to the Schizophora, since *scw* orthologues exist in aschizan cyclorrhaphan flies. In order to further localise the origin of *scw*, we have utilised new genomic resources for the nematoceran moth midge *Clogmia albipunctata* (Psychodidae). We identified the BMP subclass members *dpp* and *gbb* from an early embryonic transcriptome and show that their expression patterns in the blastoderm differ considerably from those seen in cyclorrhaphan flies. Further searches of the genome of *C. albipunctata* were unable to identify a *scw-*like *gbb* duplicate, but confirm the presence of *dpp* and *gbb*. Our phylogenetic analysis shows these to be clear orthologues of *dpp* and *gbb* from other non-cyclorrhaphan insects, with *C. albipunctata gbb* branching ancestrally to the cyclorrhaphan *gbb*/*scw* split. Furthermore, our analysis suggests that *scw* is absent from all Nematocera, including the Bibionomorpha. We conclude that the *gbb*/*scw* duplication occurred between the separation of the lineage leading to Brachycera and the origin of cyclorrhaphan flies 200–150 Ma ago.

**Electronic supplementary material:**

The online version of this article (doi:10.1007/s00427-013-0445-9) contains supplementary material, which is available to authorized users.

## Introduction

Signalling molecules belonging to the transforming growth factor β (TGFβ) group perform key roles in morphological and physiological processes in all metazoan phyla. They have been variously referred to as a superfamily or family depending on the author (Newfeld et al. 1999; Van der Zee et al. 2008). Here, we follow a commonly accepted evolutionary definition of family as a set of genes derived from a single gene present in the common ancestor of the bilaterians. Under this classification, we consider the TGFβ grouping as a class formed of two subclasses: bone morphogenetic proteins (BMPs) and the activin/TGFβ subclass (Newfeld et al. 1999). In *Drosophila melanogaster*, there are two known families of BMP-encoding genes: *decapentaplegic* (*dpp*)—a member of the vertebrate BMP2/4 family—as well as *glass bottom boat* (*gbb*) and *screw* (*scw*)—members of the BMP5/6/7/8 family (Van der Zee et al. 2008). Dpp plays multiple roles in *Drosophila* development. One of them is its key role in dorso-ventral (DV) patterning during early embryogenesis (Irish and Gelbart 1987). Scw co-operates with Dpp in this process (Arora et al. 1994). Gbb has several roles at later embryonic stages, as well as in larval and pupal development (Doctor et al. 1992; reviewed in O'Connor et al. 2006). It is weakly expressed at early stages and is not involved in DV patterning. It has been proposed that this temporal distinction might separate otherwise functionally redundant proteins (Fritsch et al. 2010).

**Fig. 1:**
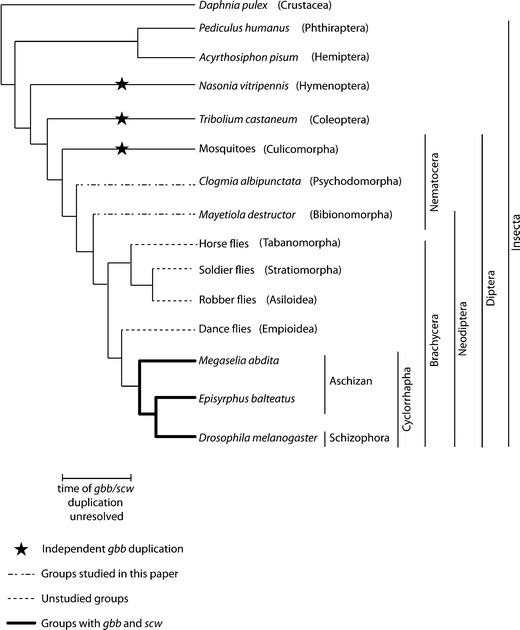
Schematic tree of organisms discussed in the text. The relationships of the class Insecta are shown including the orders Phthiraptera, Hemiptera, Hymenoptera, Coleoptera and Diptera. The Diptera are traditionally classified into two suborders, the monophyletic Brachycera and an assemblage of basally branching lineages, the Nematocera. The Nematocera include the infraorders Tipulomorpha (not shown), Culicomorpha, Psychodomorpha and Bibionomorpha. The Bibionomorpha are the likely sister group to the Brachycera and together they form the Neodiptera, whose sister group in turn is likely to be the Psychodomorpha. The Brachycera are classified into the infraorders Cyclorrhapha and the basally branching lineages of the non-cyclorrhaphan Brachycera. These include the more closely related infraorders of Asiloidea, Stratiomyomorpha and Tabanomorpha and the likely sister group of the Cyclorrhapha, the Empidoidea. The Cyclorrhapha are in turn divided into the Schizophora with the basally branching lineages of the Cyclorrhapha forming the paraphyletic assemblage of the Aschiza. Previous studies have confirmed the presence of *gbb* and *scw* in the Cyclorrhapha (*bold lines*) indicating the *gbb*/*scw* duplication occurred somewhere between the origin of this lineage and the splitting of the Psychodomorpha

Several studies have focused on the evolution of BMP-encoding genes in dipterans and other insects (Van der Zee et al. 2008; Fritsch et al. 2010; Lemke et al. 2011). While *dpp* (BMP2/4) is found in all groups studied so far, there are differences in the number and types of *gbb*-like genes (BMP5/6/7/8; see Fig. 1, for an overview of the species discussed in the text). The evidence suggests that *gbb* has undergone multiple duplications in the arthropod lineage, one of which gave rise to *scw* in the lineage leading to cyclorrhaphan Brachycera (including *D. melanogaster*; Fritsch et al. 2010). The mosquitoes *Anopheles gambiae*, *Aedes aegypti* and *Culex pipiens* (Culicomorpha) also have two closely related *gbb* genes, but these duplications appear to have occurred independently in the mosquito lineage (Fritsch et al. 2010). A similar situation applies to the flour beetle *Tribolium castaneum* (Coleoptera) with its two copies of *gbb* (*gbb1* and *gbb2*), which show distinct expression patterns, but are more closely related to each other than to any other *gbb* (Van der Zee et al. 2008). Finally, the jewel wasp *Nasonia vitripennis* (Hymenoptera) also exhibits two *gbb*-related genes, again more closely related to each other than to any other *gbb* duplicates. Arthropod species outside the holometabolan insects—such as the water flea *Daphnia pulex* (Crustacea), the human louse *Pediculus humanus* (Phthiraptera) and the pea aphid *Acyrthosiphon pisum* (Hemiptera)—have only one copy of *gbb* (Fritsch et al. 2010). According to this evidence, arthropods exhibit an ‘ancestral’ *gbb* (or *gbb1*) with high sequence conservation across lineages, and a second *gbb-*like gene in some groups (*gbb2* or *scw*), which arose by independent duplication events. It would be interesting to investigate whether the origin of the *scw* duplication can be located more precisely within the dipteran lineage.

Two recent studies have taken the first steps towards this aim. Aschizan cyclorrhaphan species, such as the hoverfly *Episyrphus balteatus* (Syrphidae), and the scuttle fly *Megaselia abdita*, have orthologs of *dpp*, *gbb* and *scw*, which show expression patterns similar to the ones in *D*. *melanogaster* (Lemke et al. 2011; Rafiqi et al. 2012). This allows us to place the duplication event giving rise to *scw* at the base of the cyclorrhaphan lineage.

We wanted to further refine the time point of the *gbb*/*scw* duplication. No lineages outside the Cyclorrhapha have been shown to have a *gbb* duplicate identifiable as an orthologue of *scw*. Here, we describe the BMP gene complement of the moth midge *Clogmia albipunctata* (Psychodidae). Despite some recent controversy over the placement of Psychodidae (Wiegmann et al. 2011), they are likely to be a sister group of Neodiptera (Brachycera plus Bibionomorpha; Yeates and Wiegmann 1999; Jimenez-Guri et al. 2013). We have found one *dpp* and one *gbb* orthologue in *C*. *albipunctata*. Using phylogenetic analysis, we have been able to group *C*. *albipunctata dpp* and *gbb* with their orthologues in other lineages. We find that *C*. *albipunctata dpp* is a clear member of the *dpp* gene family, and *C*. *albipunctata gbb* branches ancestrally to the cyclorrhaphan *gbb*/*scw* split.

## Materials and methods

### Gene identification and cloning

We searched the early embryonic transcriptome of *C*. *albipunctata* (Jimenez-Guri et al. 2013; http://diptex.crg.es) by BLAST using *dpp*, *gbb* and *scw* sequences from *D*. *melanogaster*, *M*. *abdita* and *E*. *balteatus* (retrieved from GenBank). In addition, we searched a preliminary assembly of the *C*. *albipunctata* genome (our unpublished data) and the genome of the Hessian fly *Mayetiola destructor* (Cedidomyiidae, Bibionomorpha; see Fig. 1; genome version 1.0, Baylor College of Medicine Human Genome Sequencing Center: http://www.hgsc.bcm.tmc.edu/content/hessian-fly-genome-project) with these same sequences. PCR primers for *C*. *albipunctata dpp* and *gbb* were designed from transcriptome sequences (dpp-forward CAGTAGAAGGCGTCATAACC, dpp-reverse ACGGAAAAAGAGAGTGAAAAG; gbb-forward ATCTTTATGGCAAAAGGTCTG, gbb-reverse TTTTCGAGACAAAAGAAGAAC). Amplified sequences for *C*. *albipunctata dpp* and *gbb* have been deposited in GenBank (accession numbers KC810051 and KC810052). Fragments were cloned into the PCRII-TOPO vector (Invitrogen) and used to make DIG-labelled riboprobes for in situ hybridisation.

### Whole mount in situ hybridisation

Wild-type *C*. *albipunctata* embryos were collected at blastoderm and post-gastrulation stages as described in García-Solache et al. (2010). Embryos were heat fixed using a protocol adapted from Rafiqi et al. (2011). In situ hybridisation was performed as described in Jimenez-Guri et al. (2013) and references therein.

### Phylogenetic analysis

Protein sequences of *D*. *melanogaster* Dpp, Gbb and Scw were used to identify and collect homologous sequences using the BLAST algorithm available at NCBI (http://blast.ncbi.nlm.nih.gov/Blast.cgi). The sequences were initially aligned using T-Coffee (Notredame et al. 2000). All subsequent steps (editing, re-alignment, substitution model prediction, phylogenetic analysis and tree visualisation) were carried out using the MEGA5 software (Tamura et al. 2011). Phylogenetic analyses were carried out using a maximum likelihood method and the JTT substitution model and MrBayes allowing model jumping (Huelsenbeck and Ronquist 2001; Ronquist and Huelsenbeck 2003). Online Resource 1 contains the amino acid alignment used for phylogenetic analysis in FASTA format.

## Results and discussion

### BMP subclass members in *Clogmia* include *dpp* and *gbb*, but not *scw*

The evidence described in the ‘Introduction’ section is consistent with the *gbb*/*scw* duplication occurring at the base of the Cyclorrhapha. However, it may have happened even earlier, at any time between the divergence of the culicomorph lineage and the emergence of the Cyclorrhapha (Fig. 1). In this study, we narrow the gap between mosquitoes and cyclorrhaphan flies by describing the BMP complement of the moth midge *C*. *albipunctata* (Psychodidae), a dipteran species whose lineage branches basally to the Brachycera, and thus Cyclorrhapha (Jimenez-Guri et al. 2013).

We obtained putative BMP members from the transcriptome and genome of *C*. *albipunctata* and identified their relationships by reciprocal BLAST searches. We identified single putative orthologues of *dpp* and *gbb*/*scw* from an early embryonic transcriptome (Jimenez-Guri et al. 2013; Diptex database: http://diptex.crg.es, accession numbers comp946 and comp6316). Next, we searched a preliminary genome assembly for *C*. *albipunctata* (coverage >70×, scaffold N50 ∼242 kb; our unpublished data). We identified the same single copies of the putative *dpp* and *gbb*/*scw* orthologues. No additional *gbb* duplicate is present in the genome. At this point, we cannot rule out the possibility that *C*. *albipunctata* has lost a *scw*-*like* duplicate secondarily.

### Phylogenetic reconstruction of BMP subclass members in flies

**Fig. 2:**
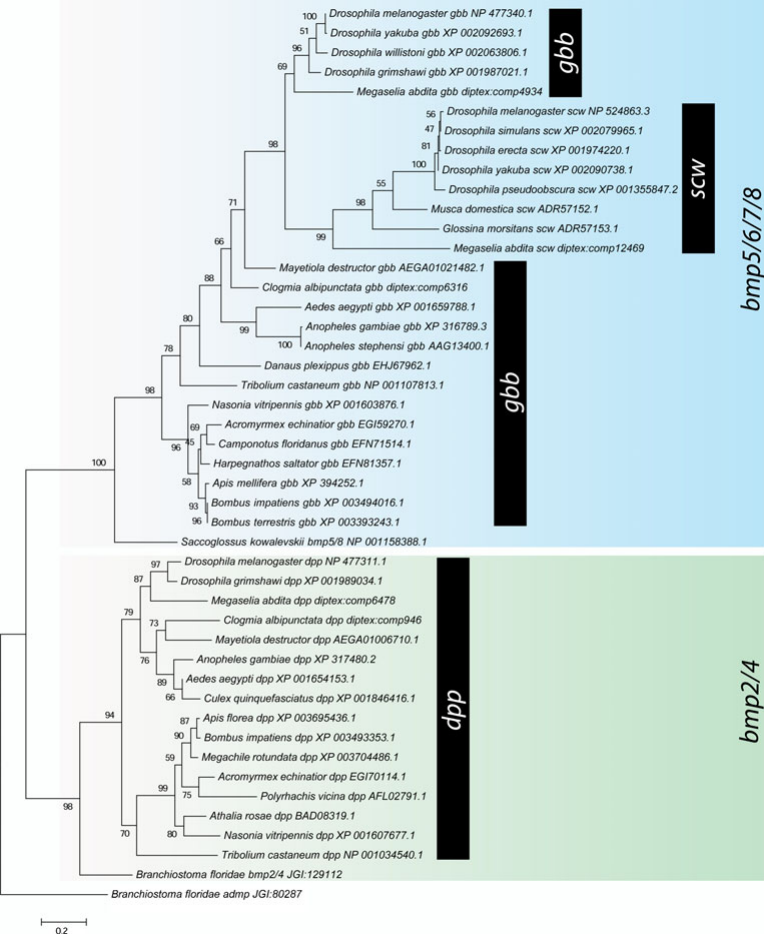
Phylogenetic analysis of *C*. *albipunctata* BMP sequences. The tree is rooted using *anti*-*dorsalizing morphogenetic protein* (*admp*) sequence from the chordate amphioxus (*Branchiostoma*). Previous analyses have suggested that *admp* belongs to a family within the BMPs outside of the BMP2/4 and BMP5678 families (Van der Zee et al. 2008). *dpp* sequences form one group with strong bootstrap support (98), with a second group consisting of *gbb*, and *gbb* duplicates, forming another strongly supported group (bootstrap, 100). Within the *dpp* group, *C*. *albipunctata dpp* branches with other dipteran sequences. The second *C*. *albipunctata* sequence branches basally to *M*. *destructor gbb*, and both branch basally to the cyclorrhaphan *gbb*/*scw* genes indicating that they did not take part in this duplication event

To test our initial classification of these sequences, and to investigate if gene loss played a role in BMP evolution, we carried out a phylogenetic analysis of the putative *C*. *albipunctata dpp* and *gbb*/*scw* sequences using maximum likelihood methods and Bayesian inference (see ‘Materials and methods’ section). Both approaches yield identical tree topologies (compare Fig. 2 with Online Resource 2). The alignment of the 192 residues used for this phylogenetic analysis can be found in Online Resource 1. Our analysis places *C*. *albipunctata dpp* and *gbb*/*scw*-like sequences into clades containing *dpp* and *gbb* from other lineages (Fig. 2). *C*. *albipunctata dpp* clusters with other dipteran *dpp* sequences, while *C*. *albipunctata gbb* branches outside of the cyclorrhaphan *gbb*/*scw* group but within the *gbb* sequences found in other arthropods. This strongly suggests an origin of *scw* after the psychodid lineage separated from the brachyceran lineage and argues against the possibility of secondary loss of *scw* in *C*. *albipunctata*. Moreover, *gbb* from the Hessian fly *M*. *destructor* (whose lineage, the Bibionomorpha, is the sister group to the Brachycera; see Fig. 1) also branches basally to the cyclorrhaphan *gbb*/*scw* group suggesting an origin of *scw* within the Brachycera.

### Expression patterns of *dpp* and *gbb* in *C*. *albipunctata*

**Fig. 3:**
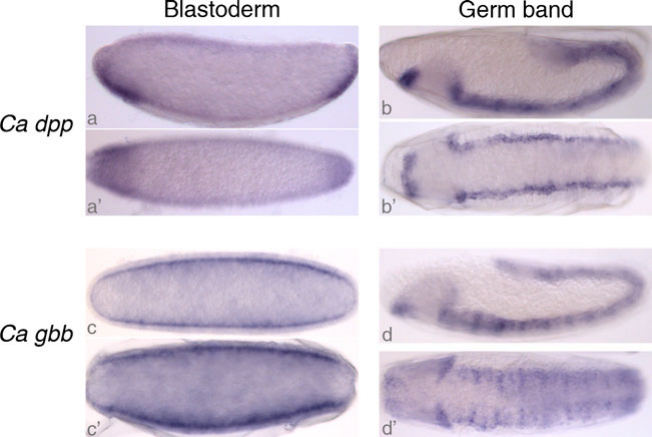
In situ hybridisation of BMP transcripts from *C*. *albipunctata*. Expression patterns for *C*. *albipunctata dpp* (*a*, *a*′, *b*, *b*′) and *gbb* (*c*, *c*′, *d*, *d*′) genes at blastoderm (*a*, *a*′, *c*, *c*′) and extended germ band stages (*b*, *b*′, *d*, *d*′). Lateral (*a*, *b*, *c*, *d*) and ventral (*a*′, *b*′, *c*′, *d*′) views are shown. Anterior is to the left. See text for a detailed description of expression patterns

We visualised the expression patterns of *C*. *albipunctata dpp* and *gbb* during early embryonic development using in situ hybridisation (Fig. 3). *C*. *albipunctata dpp* is initially expressed at the blastoderm stage (Fig. 3(a, a′)) and expression persists after gastrulation (Fig. 3(b, b′)). This temporal pattern is similar to that seen in other dipteran species (see St. Johnston and Gelbart 1987; Rafiqi et al. 2012). However, the early spatial expression pattern is very different: instead of a continuous dorsal domain as in *D*. *melanogaster* or *M*. *abdita*, or a discontinuous dorsal domain to the exclusion of the presumptive serosa as in *A*. *gambiae* (Goltsev et al. 2007), *C*. *albipunctata dpp* is expressed at the anterior and posterior end of the ventral blastoderm (Fig. 3a). This pattern is very surprising, since ventral *dpp* expression has not yet been reported in any protostome species. Its implications for DV patterning in *C*. *albipunctata* will be analysed and reported elsewhere. Later, in the elongating germ band embryo, *C*. *albipunctata dpp* expression pattern is equivalent to that of *D*. *melanogaster* or *M*. *abdita* (Fig. 3(b, b′)).

In the early blastoderm, *C*. *albipunctata gbb* is detected in a very broad central domain, which only excludes two small terminal regions at the poles of the embryo (Fig. 3(c, c′)). This pattern also differs from the expression seen in *D*. *melanogaster*, where *gbb* is not detected before gastrulation (Doctor et al. 1992), or *M*. *abdita*, where expression is detected only in the dorsal blastoderm, although it is also excluded from the pole regions (Rafiqi et al. 2012). Similar to the expression in *M*. *abdita* and *D*. *melanogaster*, we detect *C*. *albipunctata gbb* transcript at high levels in a complex, segmentally repeated pattern after gastrulation (Fig. 3(d, d′)).

In conclusion, we identified only two members of the BMP subclass of genes in the psychodid moth midge *C*. *albipunctata*: one orthologue of *dpp* and one gene related to *gbb*. Their expression patterns at blastoderm stage differ significantly from those seen in Cyclorrapha, while expression during germ band extension is equivalent in both groups. No *scw*-like *gbb* duplicate could be identified from the early embryonic transcriptome, or the genome of *C*. *albipunctata*, and no such duplicate is present in the genome of the Hessian fly (Bibionomorpha) either. Therefore, our phylogenetic analysis suggests that the *gbb*/*scw* duplication occurred within the Brachycera. Further research in non-cyclorrhaphan Brachycera (Empidoidea, Asiloidea, Stratiomyomorpha and Tabanomorpha; Wiegmann et al. 2011; see also Fig. 1) will be required to localise the precise origin of *scw* within the basal branches of the Brachycera.

## Electronic supplementary material

Below is the link to the electronic supplementary material.

Online Resource 1 Amino acid alignment used for phylogenetic analysis (FASTA format) (PDF 27 kb)

Online Resource 2 Bayesian phylogenetic analysis of BMP sequences (PDF 5,563 kb)
