# Supplementary material for: Evolution and expression of BMP genes in flies
Source: Dev Genes Evol. 2013 Apr 18;223(5):335–40. doi: 10.1007/s00427-013-0445-9 (PMC3744649; doi:10.1007/s00427-013-0445-9)
Supplement: Supplementary file 1 — Amino acid alignment used for phylogenetic analysis (FASTA format) (PDF 27 kb) [file 427_2013_445_MOESM1_ESM.pdf]

Evolution and expression of BMP genes in flies (Development Genes and Evolution) Karl R. Wotton, Anna Alcaine Colet, Johannes Jaeger, Eva Jimenez-Guri
EMBL/CRG Research Unit in Systems Biology, Centre de Regulació Genòmica (CRG), and Universitat Pompeu Fabra (UPF), Barcelona, Spain
eva.jimenez@crg.es

Electronic Supplementary Material. Online Resource 1: Amino acid alignment used for phylogenetic analysis in FASTA format

Full sequences can be downloaded from NCBI GenBank http://www.ncbi.nlm.nih.gov/genbank via accession numbers. Numbers labelled with "JGI" can be accessed from the Joint Genome Institute www.jgi.doe.gov and those with "diptex" from the Diptex server http://diptex.crg.es/

```
-------------------------------------------------------------------------------------------

>Acromyrmex_echinatior_dpp_EGI70114.1
QGRVHVPKSLRDLHAR---
QNAIGIADIRSANTVRSFPHIEFNLSRIPSGEMLQAAELSLTRRGRILVYATINLDVHPAVVRWIENHQDNHGLRPMLYTYMDGRYERARRADFADVGWNDWIVAPPGYDAFY
CHGDCPFPLADHLNSTNHAIVQTLVYSTKPTVPKACCVPTALSSISMLYLVVLKNYLGCG
>Acromyrmex_echinatior_gbb_EGI59270.1
QVKRSAPKFLLDIYKNALGEDRNRKAGIDQSDVIMTFAAHNFDVSEVPPEEYIIGAELRLYHSHTITAYGWLTLNISEAFEHWVKDSKENRGLQPFIVGYFKSSGVRQKRDSF
RDLQWQDWIIAPDGYDAYYCSGECNFPLNAHMNATNHAIVQTLVHLVSPKVPKPCCAPTKLSAISVLYFLILKKYKSCG
>Aedes_aegypti_dpp_XP_001654153.1
RSKVVIPEAMKQLYAQIMGHDLVDSVNVRNANTVRSFTHEEFNVSNIPRSEKLRAAELTLNRQHQILVYGSISVDVLPAVERWLRSPKQNHGLQPLLFTYTDGRHKRARRADF
SDVGWSDWIVAPPGYEAYYCHGDCQFPIADHLNTTNHAIVQTLVNSINPSAPKACCVPTQLSSISMLYLVVLKNYVGCG
>Aedes_aegypti_gbb_XP_001659788.1
SISKSAPQFLLNVYDKLSSDTHNRHVRIDQSDIIMTFLNKNFDVNEMEDDAELILAELRLYQNITVAVYGWIELNVTSTVMQWTTDASSNKGFQPFLVAYCRSPQISLSRSSF
KDLNWQDWIIAPEGYGAFFCSGECNFPLNAHMNATNHAIVQTLVHLMHPKVPKPCCAPTKLIPISVLYHINLKKYKSCG
>Anopheles_gambiae_dpp_XP_317480.2
RSKVVIPEAMKQLYAQIMGHDLVDSVSVRNANTVRSFTHEEFNVTSIPRGEKLRAAELTLTRLYQVMVYGTASFDVMPAVERWLRQPRKNHGLQPLLFTYTDGRHKRARRADF
SDVGWNDWIVAPPGYEAYYCQGDCRFPIADHLNTTNHAIVQTLVNSYNPTAPKACCVPTQLSSISMLYLVVLKNYVGCG
>Anopheles_gambiae_gbb_XP_316789.3
SVGKSAPQFLLNVYDQLQQEERIRKVRIDESDIIMTFLNKNFGIANIEDDVSVLMAELRLYRSLTLTVYGWLEINVTGAVNLWLKNRQANHGLQPFLVVYANSQQQRNKRSSF
KDLQWHEWIIAPEGYGAYYCSGECNFPLNAHMNATNHAIVQTLVHLNHPKVPKPCCAPTKLIPISVLYHINLKKYKSCG
>Anopheles_stephensi_gbb_AAG13400.1
SVGKSAPQFLLNVYDQLQQEERIRKVRIDESDIIMTFLNKNFGITNIEDDVSVLMAELRLYRSLTLTVYGWLEINVTGAVNLWLKNRQANHGLQPFLVVYANSQQQRNKRSSF
KDLQWHEWIIAPEGYGAYYCSGECNFPLNAHMNATNHAIVQTLVHLNHPKVPKPCCAPTKLIPISVLYHINLKKYKSCG
>Apis_florea_dpp_XP_003695436.1
QGPAYVPESLKXLFIK---
QNTIGTADIRSANTVRSFSHVEFDLSSIPSGEKLQAAELSLSRLGRLLIYGTISLDVHPAVERWIKDPKNNHGLRPMLFTYTDGRYKRARRADFADVGWNDWIVAPPGYDAFY
CHGDCPFPLADHLNSTNHAIVQNLVYSTKPSVPKACCVPTALSSISMLYLVVLKNYLGCG
>Apis_mellifera_gbb_XP_394252.1
QVKRSAPKFLLDIYKNALGEDQQRRAGIDQSDVIMTFAAHNFDVSEVPPGEHIIGAELRLYRSYMITAYGWLTLNISESLSHWVNNPDGNKGLQPFMVGYFKSSGIRQKRDSF
RDLKWQDWIIAPDGYDAYYCSGECNFPLNAHMNATNHAIVQTLVHLVSPKVPKPCCAPTKLSPISVLYFLILKKYKSCG
>Athalia_rosae_dpp_BAD08319.1
LGPSHVPESLKKLYLR---
QSASGMADIRSSNTVRSFAHVEFDLSSVPLQETLHAAELSLSRYQRILVHSSVPLDVHPAVERWIQNPSQNYGLRPFLFTYTDGRNERARRADFVDVGWNDWIVAPPGYDAFY
CHGDCPFPLADHLNSTNHAIVQTLVYSTNPNVPKACCVPTALSSISMLYLVVLKNYLGCG
>Bombus_impatiens_dpp_XP_003493353.1
QGQAYVPESLKKLFVK---
QNTIGMADIRSANTVRSFSHVEFDLSSIPSGEKLQAAELSLSRLGRILIYGTISVDVHPAVERWIKDPKNNHGLRPMLFTYTDGRYKRARRADFADVGWNDWIVAPPGYDAFY
CHGDCPFPLADHLNSTNHAIVQNLVYSTKPSVPKACCVPTALSSISMLYLVVLKNYLGCG
>Bombus_impatiens_gbb_XP_003494016.1
QVKRSAPKFLLDIYKNALGEDQQRRAGIDQSDVIMTFAAHNFDVSEVPPGEHIISAELRLYRSYMITAYGWLTLNVSEPLDHWVNNPDGNKGLQPFMVGYFKSSGIRQKRDSF
RDLKWQDWIIAPDGYDAYYCSGECNFPLNAHMNATNHAIVQTLVHLVSPKVPKPCCAPTKLSPISVLYFLILKKYKSCG
>Bombus_terrestris_gbb_XP_003393243.1
QVKRSAPKFLLDIYKNALGEDQQRRAGIDQSDVIMTFAAHNFDVSEVPPGEHIISAELRLYRSYMITAYGWLTLNVSEPLDHWVNNPDGNKGLQPFMVGYFKSSGIRQKRDSF
RDLKWQDWIIAPDGYDAYYCSGECNFPLNAHMNATNHAIVQTLVHLVSPKVPKPCCAPTKLSPISVLYFLILKKYKSCG
>Branchiostoma_floridae_admp_JGI_80287
KRHFRPPQYMLDLYNTISADGIVRFPNPLSANVVRSFPDKDFNVSSVSVTENVLDAELHLFKFYEVRVYGWEVFNIKPAVQDWVADKNANFGLEPILVLFSDG---
RPRSADFDAIGWSGWIISPKGYNAYHCKGKCPFPLGQSQKPTNHATVQSIMNALRQEVGQPCCVPNKLYSINLLYFVILKQYANCG
>Branchiostoma_floridae_bmp2/4_JGI_129112
RKNLVIPPYMLELYLSQTKDPNFNFAGTSTANTVRSFHHEEFNTSAVPSVELIKAAELRLFRLYRVNVYSWESFDVRSAVTKWKNSPERNYGLRPLLLTYTDGKG-
RQKRADFSDVGWNDWIVAPPGYQAYYCHGECPFPLADHLNSTNHAIVQTLVNSVNPAVPKACCVPTDLSPISMLYLNVLKNYEGCG
>Camponotus_floridanus_gbb_EFN71514.1
QVKRSAPKFLLDIYKNALGEDHHRKAGIDQSDVIMTFAAHNFDVSEVPPQEYIIGAELRLYRSHTITVYGWLTLNVTGALDHWVNNPDENRGLQPFMVGYFKSSGIRQKRDSF
RDLQWQDWIIAPDGYDAYYCSGECNFPLNAHMNATNHAIVQTLVHLLNPKVPKPCCAPTKLSAISVLYFLILKKYKSCG
>Clogmia_albipunctata_dpp_diptex:comp946
RSKVVIPEELKQLYAQLTGEDLDLVNVPKSANTVRSFTHEDFNVSSIPKDEHLKVAELILSRRHTVQVYAPIKLDIMPAVERWRRTPTENHGVQPLLFTYTDERHRRPRRADF
QDVGWSDWIVAPLGYDAFYCKGECHLPLSDHLNSTNHAVVQTLVNSFDSHAPRACCVPTQLTSISMLYLVVLKNYVGCG
>Clogmia_albipunctata_gbb_diptex:comp6316
SLRKSAPQFLLDIYRRLAEEDSPRSTRIDQSDIIMTFLNKNFDVSEVSKELSLMMAELRVYQIFTISVYGWLELNVTGALSRWIGNLPQNRGLQPFMVGFFKGQDHRTKRNSF
KDLKWQDWIIAPDGYGAYFCSGECNFPLNSHMNATNHAIVQTLVHLLHPKVPKPCCAPTKLSAISVLFFINLKKYKSCG
>Culex_quinquefasciatus_dpp_XP_001846416.1
RSKVVIPEAMKQLYAQIMGHELDDSVNVRNANTVRSFTHEEFNVTNIPLNEKLRAAELTLNRVHQILVYAPISVDVLPAVDRWMRNPKQNHGLQPLLFTYTDGRHKRARRTDF
```

SDVGWSDWIVAPPGYEAFYCQGDCQFPIADHLNTTNHAIVQTLVNSISPSAPKACCVPTQLSSISMLYLVVLKNYVGCG
>Danaus_plexippus_gbb_EHJ67962.1
PLDRSAPSFLLDVYKQLAEEHRPTRSSIDESDLIMTFQSKKFDVASAPGDSSLLAAELRLHQLYTVVAHGWLEFNVTSALATWLGAPADNNGFQPFMVAFFKGPK-
RKKRESFKDLEWQDWIIAPDGYGAFYCSGECNFPLNAHKNATNHAIVQTLVHLLNPQVPKPSCAPIKLSPISVLYYTILRKYKSCG
>Drosophila_erecta_scw_XP_001974220.1
NLHNSASKFLLEVYNEIEDQRHKRSLDDRQEISILTFSSREFNTNDVPVDLSLVQAMLRIYKQFTVSVYGWLEFNLTETLRSWLLISIGDAGLEPFIVGYFNGPELRFKRSDF
KELHMHNWVIAPKKFEAYFCGGGCNFPLGTKMNATNHAIVQTLMHLKQPHLPKPCCVPTILGAITILRYLNLTKYKECG
>Drosophila_grimshawi_dpp_XP_001989034.1
RSKIVIPEAMKKLYAQIMG-
HELDSVNIKSANTVRSFTHKDFDVKSIPADEKLKAAELQLTRRYQVLVYDTVSLDVQPAVDRWLATPHKNYGLQPLLFTYTDGRHKRNRRHDFQDVGWSDWIVAPPGYDAFYC
HGKCPFPLADHLNSTNHAVVQTLVNNLNPGVPKACCVPTQLEGISMLYLVVLKNYVGCG
>Drosophila_grimshawi_gbb_XP_001987021.1
SLRKSAPKFLLDVYNRIEEGRSKRDADDKRAIIIMTFLNKEFDVTNVPGDNYLMMSELRIYQNFTVTVYGWLELNVTEALHDWLTIGAHDIGLQPFMIGFFRGPELRTKRTDF
KDLGWHDWIIAPDGYGAFYCSGECNFPLNAHMNATNHAIVQTLVHLLEPKVPKPCCAPTKLGALPVLYHLNLKKYKSCG
>Drosophila_melanogaster_dpp_NP_477311.1
RSKIIIPEPMKKLYAEIMG-
HELDSVNIKSANTVRSFTHKDFDVKSIPADEKLKAAELQLTRRYQVLVYDTVSLDVQPAVDRWLASPQRNYGLQPLLFTYTDGRHKRNKRQDFSDVGWDDWIVAPLGYDAYYC
HGKCPFPLADHFNSTNHAVVQTLVNNMNPGVPKACCVPTQLDSVAMLYLVVLKNYVGCG
>Drosophila_melanogaster_gbb_NP_477340.1
SLRKSAPKFLLDVYHRIQDERSRRSADDKRAIIIMTFLNKDFDVSNVPNDNYLVMAELRIYQNFTITVYGWLELNVTEGLHEWLVIGAHDIGLQPFMIGFFRGPELRSKRSDF
KDLGWHDWIIAPEGYGAFYCSGECNFPLNAHMNATNHAIVQTLVHLLEPKVPKPCCAPTRLGALPVLYHLNLKKYKSCG
>Drosophila_melanogaster_scw_NP_524863.3
NLHNSASKFLLEVYNEIEDQRHKRSLDDRQEISILTFSSREFNTNDVPVDLSLVQAMLRIYKQFTVSVYGWLEFNLTDTLRYWLHISIGDAGLEPFIVGYFNGPELRFKRSDF
KELHMHNWVIAPKKFEAYFCGGGCNFPLGTKMNATNHAIVQTLMHLKQPHLPKPCCVPTVLGAITILRYLNLTKYKECG
>Drosophila_pseudoobscura_scw_XP_001355847.2
NLHNSASKFLLEVYNEIEGQRHKRSLDDRQEISILTFSSREFNTNDVPADLTLVQAMLRVYKQMTVSVYGWLEFNLTPTLRTWLFFSIGDAGLEPFIVGYFNGPELRFKRSDF
KELQMHNWVIAPKKFEAYFCGGGCNFPLGTKMNATNHAIVQTLMHLKQPHLPKPCCVPTVLGSITILRYLSLTKYKECG
>Drosophila_simulans_scw_XP_002079965.1
NLHNSASKFLLEVYNEIEDQRHKRSLDDRQEISILTFSSREFNTNDVPVDLSLVQAMLRIYKQFTVSVYGWLEFNLTDTLRSWLLISIGDAGLEPFIVGYFNGPELRFKRSDF
KELHMHNWVIAPKKFEAYFCGGGCNFPLGTKMNATNHAIVQTLMHLKQPHLPKPCCVPTVLGAITILRYLNLTKYKECG
>Drosophila_willistoni_gbb_XP_002063806.1
SLRKSAPKFLLDVYHRIANQRSKRDADDKQAIIIMTFLNKDFDVSNVPSDNSLMMAELRIYQDFTISVYGWLELNVTEGLHDWLVIGAHDIGLQPFMIGFFRGPELRAKRSDF
KDLGWHDWIIAPEGYGAFYCSGECSFPLNAHMNATNHAIVQTLVHLMEPKVPKPCCAPTRLGALPVLYHLNLKKYKSCG
>Drosophila_yakuba_gbb_XP_002092693.1
SLRKSAPKFLLDVYHRIQDERARRSADDKRAIIIMTFLNKDFDVSNVPNDNYLVMAELRIYQNFTITVYGWLELNVTEGLHEWLVIGAHDIGLQPFMIGFFRGPELRSKRSDF
KDLGWHDWIIAPEGYGAFYCSGECNFPLNAHMNATNHAIVQTLVHLLEPKVPKPCCAPTRLGALPVLYHLNLKKYKSCG
>Drosophila_yakuba_scw_XP_002090738.1
NLHNSASKFLLEVYNEIEDQRHKRSLDDRQEISILTFSSREFNTNDVPVDLSLVQAMLRIYKQFTVSVYGWLEFNLTETLRTWLLISIGDAGLEPFIVGYFNGPELRFKRSDF
KELHMHNWVIAPKKFEAYFCGGGCNFPLGTKMNATNHAIVQTLMHLKQPHLPKPCCVPTVLGAITILRYLNLTKYKECG
>Glossina_morsitans_scw_ADR57153.1
PLKNSAAKFLLEVYNIVVQQRSKRSIRDVMEITIIITYPSKGFATNRVSNDLQLVHSVLRIYQQATVFVYGWLEFDITETLHKWLKIAVNGLGLQPFIIAYFNGPELRTKRSDF
EDLNMGEWIIAPKRYEAYFCAGECSFPLNMQTDATNHAIVQNLMNIKRPSLPKPCCTPVLLSSIKILHYDILTKFKGCG
>Harpegnathos_saltator_gbb_EFN81357.1
QVKRSAPKFLLDIYKNALGEDHRRKAGIDQSDVIMTFALHNFNVSEVPPGEHIIAAELRLYRSYTITAYGWLSLNVSEALEHWVNNPDGNRGLQPFLVGYFKSSGKRQKRDSF
RDLQWQDWIIAPDGYDAYYCSGECNFPLNAHMNATNHAIVQTLVHLVSPKVPKPCCAPTKLSAISVLYFLILKKYKSCG
>Mayetiola_destructor_dpp_AEGA01006710.1
RSRIVIPEAMKALYSEIMGEELRESVNLKSANTVRSFLHEEFNVSGIPKDEILKAAELELQRRHKVQVFEAICLDVTPAVERWLDKPKQNYGIQPTLMTYTDGRYKRGRRADF
SNVGWSDWIVAPGGYDAYYCHGECHFPFADHLNTTNHAVVQTLVNSINSDAPKACCIPTQLKPISMLYLVVLKSYVGCG
>Mayetiola_destructor_gbb_AEGA01021482.1
SLRKSAPKFLLDVYKKLNDEAYERSTDIEQSDIITTFLNKKFDTSEVSAESTLMMSELRIFQEFIITIYGWLELNVTDAMSKWIADSSTNRGLQPFMIGYFKGQNFRTKRSSF
KDLKWQDWIIAPDGYGAFYCSGECNFPLTAHMNATNHAIVQTLVHLMQPKVPKPCCAPTKLSPISVLYYLNLKKYKSCG
>Megachile_rotundata_dpp_XP_003704486.1
QGPAHVPESLKKLYIK---
QNTIGMADIRSANTVRSFQHIEFDLSSIPSGEKLQAAELSLSRLSRILVYDTVSLDVHPAVERWMNDPKNNHGLRPMLFTYTDGRYKRARRADFADVGWNDWIVAPPGYDAFY
CHGDCPFPLADHLNSTNHAIVQNLVYSTKPSVPKACCVPTALSSISMLYLVVLKNYLGCG
>Megaselia_abdita_dpp_diptex:comp6478
RSKIVIPEAMKKLYAEIMG-
QELDSINIKTANTVRSFTHQEFNLSSIPEDEKLKAAELQINRRYQVFVYDILSFDVQPAVERWLATPKANHGLQPILYTYTDERFTRTKRQDFSEVGWSDWIVAPNGYDAFYC
GGECPKIMSVEFNTTNHAILQQMINQNNPEVPKACCVPTQLAPISMLYLVVLKSYVGCG
>Megaselia_abdita_gbb_diptex:comp4934_c0_seq1_6
SLRKSAPQFLLDIYHRIIENRSKRDLNDKKAAVIMTFLNKGFDTNDVETDNHLLSAEFRIYQNFVISVHGWLEVNVTEALHQWSYISAHGIGLQPFMIGFFKGPELRNKRNDF
NDLKWKDWIVAPDGYAAYYCSGECEFPLNTHMNATNHAIVQTLVHLTQPKVPKACCVPSSLGLLPVLYHQTLKKYKSCG
>Megaselia_abdita_scw_diptex:comp12469
SLRRSSVKYLVDVYNSLEEDTKRRYTRAGEHINIVTFSSKTFNLTGIASDYFLTKAEIRLFQTFTIAVWGWMQLDVTASLAKALKISVHGFGIQPFLIGFFDGPDSRQKRQHV
KDLDMDEIILAPKTFDAYFCSGECNFPLQAQMNATNHALIQTLAHLKNPSIPKPSCSPRTLGSIRVLEYLNLKPYKTCG
>Musca_domestica_scw_ADR57152.1
SLKNSASKFLLEVYNDILDHRQRRALRDRMEINIITFSSKAFNTNDVPRDLQLAHSALRLYQQAIISIYGWLEFNMTRILSRWLQVGVTSLGLQPFIIGYFNGPELRFKRSDF
NDLQMHDWVIAPKKFEAFFCGGECNFPLGSKMNATNHAIVQTLMHLKQPNLPKPCCVPTVLGSISILHYLNLSKYKECG
>Nasonia_vitripennis_dpp_XP_001607677.1
SGQTHVPEQLKQLYRR---
QSASGVVDIGPANTVRSFLHVEFDVSSVPAGERLQAAELSLSRLVRLMVHASVSLDVRPALERWISKPNQNHGLRPTLYAYTDGRKKRARRADFADVGWNDWIVAPPGYDAFY
CHGDCPFPLADHLNSTNHAIVQNLVYSTNPSVPKACCVPTSLGSISMLYLVVLKNYLGCG
>Nasonia_vitripennis_gbb_XP_001603876.1
QVKRSAPKFLLDIYRNGLADEHQTEFGIDQSDVIMTFAAHNFDVSEVPENEHMINAELRLYRAFTLTAYGWLSLNVSEALQYWVANPAGNRGLQPFMVAFFKSSGIRQKRDSF
RDLQWQDWIIAPDGYDAYYCSGECNFPLNAHMNATNHAIVQTLVHLVNPKVPKPCCAPTKLSPISVLYFLILKKYKSCG
>Polyrhachis_vicina_dpp_AFL02791.1

QLKRMSPLLSLVLVAAIANSNRPKPQGTADANVVRSFYHVEFDLSKVPNEETLQASELLLSR--RVLVYATVSLDVHPAVVRWIQNHRDNHGLQPMLFAYMDGRY-
RARRADFADVGWNDWIVAPPGYDAFYCHGDCPFPLADHLNTTNHAIVQTLVYSTKPMVPKACCVPTALSSISMLYLDVLKNYLGCG
>Saccoglossus_kowalevskii_bmp5/8_NP_001158388.1
ATHNSAPKFMIDLYRTIXTENNETLEDIDNADVVMSFPLHQFDVSDVSLEDTVTSAELRMYKSYKIHVYGWLVFDVTHAVDVWDLLEKDKLKIQPFVVAFFKTSEERRDRSSF
RELGWQDWIIAPDGYSAFYCNGECSFPLNAHMNATNHAIVQTLVHLMDPEVPKPCCAPTKLNAISVLYFVILKKYKSCG
>Tribolium_castaneum_dpp_NP_001034540.1
EGKVQVPEALKKIYNIQN-NFSLPLPGTKSANTIRSFTHVEFNISSIPRHEKLTAAEIKLTRFQRVLVHTTVSIDVFPAVARWMQDPKTNHGIQPLLFTYTDGKN-
RPKRQDFGSVGWNDWIVAPLGYDAYYCGGECEYPIPDHMNTTNHAIVQSLVNSMKPEVPGPCCVPTQLGQMSMLYLGILKNYVGCG
>Tribolium_castaneum_gbb_NP_001107813.1
SLKRSAPKFLLDIYKSLMEEERSERSAIDESDVIMTFESINFNVSEMPIAENVVGAELRIYQVYTVTVFGWLNLNLTACLPTWVAFPDSNKGLQPFMVAFLKASNHRDLSSSF
KDLKWQDWIIAPAGYSAHYCAGECKFPLNGHMNATNHAIVQTLVHLMYPKYPKPCCAPTKLTPISVLYFQILKKYKSCG
