# Supplementary material for: Evolution and expression of BMP genes in flies
Source: Dev Genes Evol. 2013 Apr 18;223(5):335–40. doi: 10.1007/s00427-013-0445-9 (PMC3744649; doi:10.1007/s00427-013-0445-9)

Evolution and expression of BMP genes in flies (Development Genes and Evolution)
Karl R. Wotton, Anna Alcaine Colet, Johannes Jaeger, Eva Jimenez-Guri
EMBL/CRG Research Unit in Systems Biology, Centre de Regulació Genòmica (CRG),
and Universitat Pompeu Fabra (UPF), Barcelona, Spain
eva.jimenez@crg.es

Electronic Supplementary Material. Online Resource 2:
Bayesian phylogenetic analysis of BMP sequences

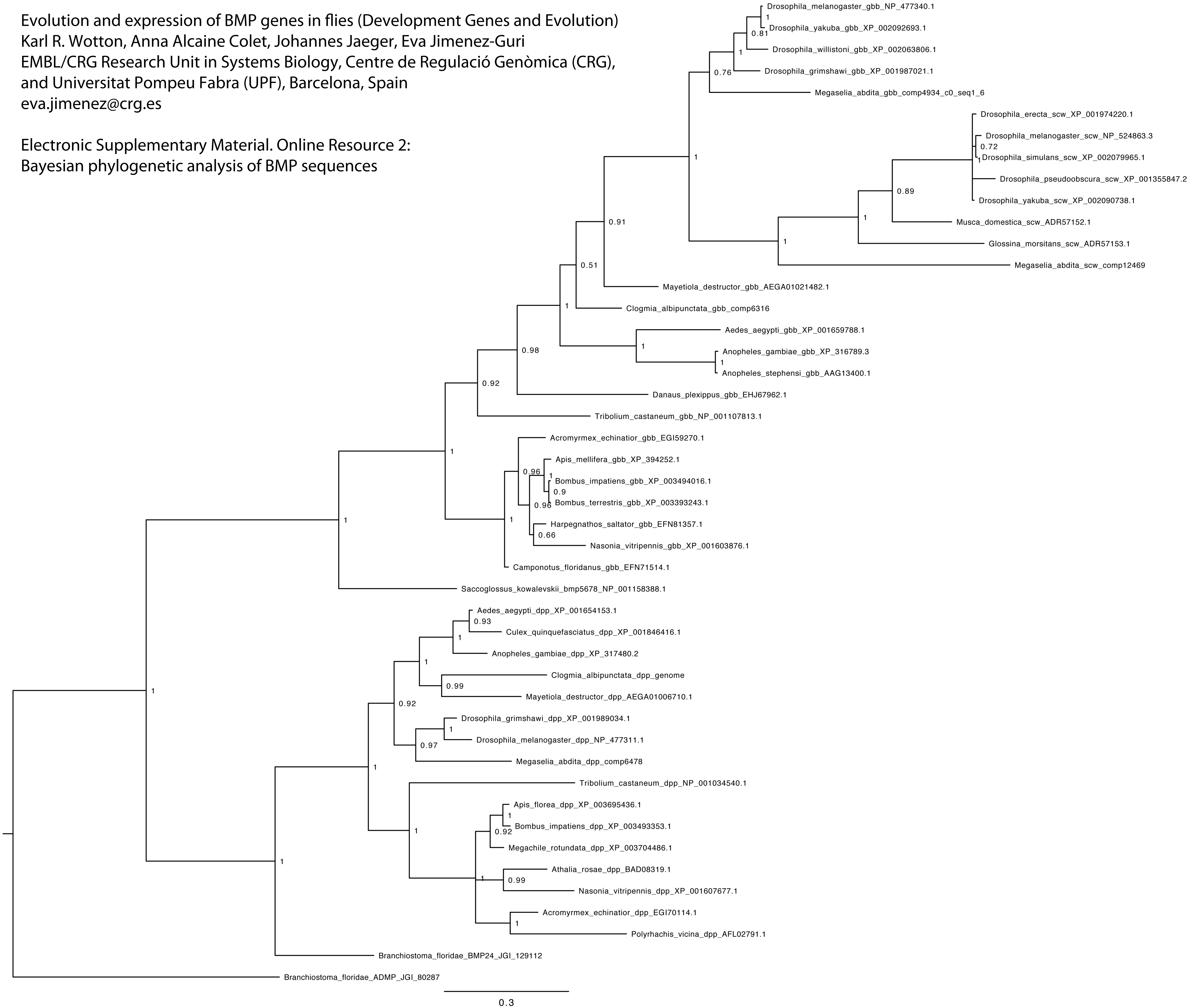

Supplement: Supplementary file 2 — Bayesian phylogenetic analysis of BMP sequences (PDF 5,563 kb) [file 427_2013_445_MOESM2_ESM.pdf]
